# Supplementary material for: Machine learning model based on radiomics features for AO/OTA classification of pelvic fractures on pelvic radiographs
Source: PLoS One. 2024 May 30;19(5):e0304350. doi: 10.1371/journal.pone.0304350 (PMC11139281; doi:10.1371/journal.pone.0304350)
Supplement: Attachment — Submitted filename: Response to Reviewers.pdf [file pone.0304350.s001.pdf]

Editor

1. Please ensure that your manuscript meets PLOS ONE's style requirements, including those for file naming. The PLOS ONE style templates can be found at https://journals.plos.org/plosone/s/file?id=wjVg/PLOSOne_formatting_sample_main_body.pdf and https://journals.plos.org/plosone/s/file?id=ba62/PLOSOne_formatting_sample_title_authors_affiliations.pdf.

Answer

As the editor mentioned, it appears that the format of this paper did not fit the requirements defined by PLOS ONE. Therefore, the authors revised and supplemented the overall format of the paper by referring to the PDF files mentioned by the editor in the email. Thanks to the editor's comments, the completeness of the paper has improved. Thank you.

2. Please note that PLOS ONE has specific guidelines on code sharing for submissions in which author-generated code underpins the findings in the manuscript. In these cases, all author-generated code must be made available without restrictions upon publication of the work. Please review our guidelines at https://journals.plos.org/plosone/s/materials-and-software-sharing#loc-sharing-code and ensure that your code is shared in a way that follows best practice and facilitates reproducibility and reuse.

Answer

As the editor mentioned, we believe there was a lack of submissions supporting the results of this study. Evidence that supports research results is essential and can improve the reliability of experimental results. Therefore, the authors uploaded the entire code and virtual environment used in the experiment to GitHub. Please refer to the path below.
https://github.com/user-dynamite/biomedical-engineering/tree/master

3. Thank you for stating the following financial disclosure: "This work was supported by the Korea Medical Device Development Fund grant funded by the Korea government (the Ministry of Science and ICT, the Ministry of Trade, Industry and Energy, the Ministry of Health & Welfare, the Ministry of Food and Drug Safety) (Project Number: 1711196789, RS-2023-00252804)."
Please state what role the funders took in the study. If the funders had no role, please state: ""The funders had no role in study design, data collection and analysis, decision to publish, or preparation of the manuscript.""
If this statement is not correct you must amend it as needed.
Please include this amended Role of Funder statement in your cover letter; we will change the online submission form on your behalf.

Answer

Thank you for providing funding and for carefully reviewing and confirming the details of the paper. The funders who provided funding for this paper had no role in the preparation of the manuscript. A description of the role of the funder was included in the cover letter. Please confirm.

4. Thank you for stating the following in the Acknowledgments Section of your manuscript: "This work was supported by the Korea Medical Device Development Fund grant funded by the Korea government (the Ministry of Science and ICT, the Ministry of Trade, Industry and Energy, the Ministry of Health & Welfare, the Ministry of Food and Drug Safety) (Project Number: 1711196789, RS-2023-00252804)."
We note that you have provided funding information that is not currently declared in your Funding Statement. However, funding information should not appear in the Acknowledgments section or other areas of your manuscript. We will only publish funding information present in the Funding Statement section of the online submission form.
Please remove any funding-related text from the manuscript and let us know how you would like to update your Funding Statement. Currently, your Funding Statement reads as follows: "This work was supported by the Korea Medical Device Development Fund grant funded by the Korea government (the Ministry of Science and ICT, the Ministry of Trade, Industry and Energy, the Ministry of Health & Welfare, the Ministry of Food and Drug Safety) (Project Number: 1711196789, RS-2023-00252804)."

Please include your amended statements within your cover letter; we will change the online submission form on your behalf.

Answer

We are fully aware of the failure to carefully check the details of the funds statement in the PLOS ONE form. Thanks to the Editor's comments, we were able to check and review the matter in detail. All text related to funds was deleted from the manuscript. The contents of the fund statement were revised and supplemented and written in the cover letter.

5. In the online submission form, you indicated that the data used to support the findings of this study are available upon request from the corresponding authors.
All PLOS journals now require all data underlying the findings described in their manuscript to be freely available to other researchers, either 1. In a public repository, 2. Within the manuscript itself, or 3. Uploaded as supplementary information.
This policy applies to all data except where public deposition would breach compliance with the protocol approved by your research ethics board. If your data cannot be made publicly available for ethical or legal reasons (e.g., public availability would compromise patient privacy), please explain your reasons on resubmission and your exemption request will be escalated for approval.

Answer

Sharing data used in research, such as the editor's comments, increases the possibility of further research and future development. This research team is fully aware of the content and believes it is important. However, it is impossible for Gachon University Gil Hospital, which received the patient data, to disclose the patient data in a public repository. If you contact the corresponding author individually, the corresponding author can share data personally with institutional approval. We ask for the Editor’s generous understanding regarding this content.

Reviewer 1

The paper titled "Machine Learning Model Based on Radiomics Features for AO/OTA Classification of Pelvic Fractures on Pelvic Radiographs" presents a study aimed at developing a machine-learning algorithm based on radiomics for quick diagnosis and classification of pelvic fractures in X-ray images. The study involved analyzing pelvic anteroposterior radiographs from 990 adults with pelvic fractures and 200 normal subjects. A total of 93 features were extracted and analyzed using various machine learning models and feature selection methods.

1. The authors state the aim is to develop radiomics-based machine-learning algorithm to rapidly diagnose fractures in pelvic X- ray images and classify pelvic instability.
The results of the study do not support this aim.

Answer

As the reviewer commented, this paper developed a radiomics-based machine learning learning model and achieved a performance of about AUC 0.75. In this paper, pelvic instability was expressed in quantitative numbers based on the features selected as important. Recent studies have achieved AO/OTA pelvic fracture classification performance of up to Sensitivity 75.7%, Precision 83.6%, and Accuracy 85.0% using ResNeXt50, RNN, and 3D-ResNet50 in CT images [1]. By using Inception-V3 on in this way, the type of fracture was classified by learning a deep learning model, but it was not expressed in quantitative numbers. They did not perform any better than medical specialists. The field is developing artificial intelligence models in various aspects to assist medical professionals in their diagnosis. The authors also believe that if additional research is conducted based on the selected characteristics, it will be able to assist medical professionals in their diagnosis. Thank you for mentioning important points to improve the completeness of this paper.

References

[1] Dreizin, David, et al. "An automated deep learning method for tile AO/OTA pelvic fracture severity grading from trauma whole-body CT." Journal of Digital Imaging 34 (2021): 53-65.

[2] Lee, Changhwan, et al. "Classification of femur fracture in pelvic X-ray images using meta-learned deep neural network." Scientific reports 10.1 (2020): 13694.

2. How was the ROI defined, the extent and the software used?

Answer

As the reviewer commented, I felt that this paper lacked mention of this topic. In this study, the ROI corresponding to the pelvic region was defined by referring to the findings of pelvic AP X-rays and CT images by a trauma surgeon with more than 10 years of experience. Additionally, the defined ROI includes the left and right ilium, pubic bone, and ischium. The software used to obtain ROI was the commercial software AVIEW (Corelinesoft, Seoul, Republic of Korea). This content has also been added to the paper. Thank you for leaving a comment to improve the completeness of this paper.

Modified and supplemented contents - Page 5, line 3~7

3. There are 3 classes described and ROC is provided as the metric for model performance. Are the results supporting One vs Rest or One vs One class?

Answer

This paper classified the models into four classes (Type A, Type B, Type C, Normal) and compared the performance between models by obtaining AUC values from the ROC curve. The results of this study support One vs Rest. One vs Rest turns a multi-class problem into several binary classification problems, and a binary classifier is learned by treating one class as 1 and the remaining classes as 0. This method can achieve higher performance than One vs One. In the case of One vs One, the data is split into two different classes and trained with a binary classifier. This means that as the number of classes increases, the classifier increases and becomes more sensitive to imbalances between classes. We chose One vs Rest to achieve higher performance with fewer classifiers. Added information about using One vs Rest. Thank you for improving the completeness of this paper by leaving a comment regarding the shortcomings of this paper.

Modified and supplemented contents - Page 7, line 24~26

4. How was the radiomics models better than the clinician's decision?

Answer

This paper conducted a study to classify pelvic instability using a model learned based on radiomics. The results provided in this paper do not compare or analyze the decisions of medical experts and the model's predicted results. Conventional medical specialists had standards for classifying pelvic instability, but there were no quantitative figures for instability classes. In the results of this paper, 10 radiomics features were selected as relatively important features, as shown in Figure 8. Through the results of the study, we were able to obtain quantitative figures for the class. We also found that the research results were highly reproducible. If we study multi-center data and conduct additional research in the future, we believe it will be at a level that can assist medical specialists in their diagnosis.

5. Please do a thorough check before submission that only tables and figures included are cited appropriately.

Answer

All tables and figures included in this paper are cited to support the content of the paper. Figure 1 is a pelvic AP X-ray image and an ROI mask image designating the pelvic region. Figure 2 shows an actual AP X-ray image of the pelvis where the pelvic area is not clearly visible due to organs or gas. These cases were excluded because they could be a hindrance when extracting radiomics features. Figure 3 is the result of performing the Histogram Equalization algorithm to make somewhat blurry pelvic AP X-ray images clear. The horizontal axis of the graph below in Figure 3 represents the contrast value of the image, and the vertical axis represents the frequency with which that contrast value is used. Figure 4 shows a flow chart for extracting radiomics features of the pelvic region from pelvic AP X-ray images. A total of 93 radiomics features were extracted. Figure 5 includes the statistical analysis of the machine learning model prediction results from the feature selection method algorithm after radiomics feature extraction. Figure 6 is a Heatmap graph obtained by obtaining the AUC of each combination using 7 machine learning models and 4 feature selection method algorithms. You can check the AUC of the combination in color and number at once. Figure 7 is the ROC curve for each combination of RFE feature selection method and each machine learning model. The ROC curve was obtained by selecting only the RFE feature selection method because RFE showed relatively high performance compared to other feature selection methods. Figure 8 shows the average feature importance value of each machine learning model combination for the 10 features extracted using the RFE feature selection method. Figure 9 shows the feature importance values of each machine learning model combination for the 10 features extracted using the RFE feature selection method. Table 1 shows pelvic AP X-ray images obtained from 2015 to 2020 by type. Table 2 is the distribution of Train set and Test set for each type. There are a total of 11 figures and tables,

which may seem a bit large, but they are all written to support the content of the paper. Thanks to the reviewer's comments, we were able to thoroughly check and review the figures and tables cited in the paper. The completeness of the paper has been improved thanks to the reviewer's comments. Thank you.

Reviewer 2

This article describes the performance of machine learning models using radiomic features in classifying pelvic fractures on radiographs. The model using RFE method of feature selection and SVM classifier performed with the highest AUC of 0.75 +/- 0.06. Most models using RFE feature selection performed with AUC between 0.73 and 0.74, other methods of feature selection performed with variable AUC between 0.59 and 0.73. The importance values of features were also described.

It is the first study to evaluate the performance of machine learning models in classification of pelvic fractures and describe the significant radiomics features. Other studies have used machine learning models to classify hip fractures with high accuracy.

Answer

First, thank you very much for leaving a positive comment on this paper. In this paper, we developed a radiomics-based machine learning model to classify pelvic instability. Using the prediction results, performance was evaluated and important radiomics characteristics were analyzed and explained. As the reviewer commented, our researchers believe that this is the first study to classify pelvic instability based on radiomics features. I think it is meaningful as a first-of-its-kind study, and I think the extracted important radiomics features can be used as biomarkers in the future. Thank you.
