# Supplementary material for: Machine learning model based on radiomics features for AO/OTA classification of pelvic fractures on pelvic radiographs
Source: PLoS One. 2024 May 30;19(5):e0304350. doi: 10.1371/journal.pone.0304350 (PMC11139281; doi:10.1371/journal.pone.0304350)
Supplement: Attachment — Submitted filename: Response to Reviewers.pdf [file pone.0304350.s002.pdf]

Journal Requirements

1. Please review your reference list to ensure that it is complete and correct. If you have cited papers that have been retracted, please include the rationale for doing so in the manuscript text, or remove these references and replace them with relevant current references. Any changes to the reference list should be mentioned in the rebuttal letter that accompanies your revised manuscript. If you need to cite a retracted article, indicate the article's retracted status in the References list and also include a citation and full reference for the retraction notice.

Answer

It is important to write references completely and accurately as required by the journal. We, the authors, fully agree with the journal's requirements, as the references are what support the research in the paper. We have checked and reviewed them thoroughly. We have reviewed the references of the papers and none of them have been retracted. Thank you for your comments to improve the quality of the paper.

Additional Editor Comments

1. A well written article which has clarified its methodology in response to the reviewers. A further refinement is a broader discussion of the literature and a tabulated as well as written summary of existing radiomic features and key pelvic fracture findings should be provided to authors to further strengthen the authors' findings.

Answer

First, thank you for your positive comments on this paper. Thanks to the editors and reviewers, the quality of the paper seems to have improved a lot. Additionally, as the editor comment, a more extensive discussion of the paper and existing radiomics features and findings of major pelvic fractures would have strengthened the results. We completely agree with the editor's opinion. We have included a full discussion of the paper and a summarized version of the broader results in the final paragraph of Results and Discussion. We believe that it is essential to define the presentation of preexisting pelvic fractures. Pelvic fracture classification was added to the data section of the Materials and Methods section, as shown in Table 1. Because this is the basis from which we got our data. Table 1 is based on reference [12]. However, in our review, previous studies have not found radiographic features to classify pelvic fracture type. Thank you for mentioning important points to improve the paper.

Modified and supplemented contents - Page 11, line 25~32 / Page 4, line 16~19
